# Proceedings of the Oregon State Dental Society

**Published:** 1873-08

**Authors:** J. R. Cardwell

**Affiliations:** Salem


### PROCEEDINGS OF THE OREGON STATE DENTAL SOCIETY.

### SECOND ANNUAL SESSION.

SALEM, Wednesday, June 25, 1873.

The Oregon State Dental Society met in the City Council Chamber at 10 o'clock, and was called to order by Dr. L. S. Skiff, President.

The session opened with prayer by Dr. Gray, of Albany. The reading of minutes of the last meeting, report of various committees and officers, were deferred until the afternoon session, in order to accommodate certain members to arrive on the incoming trains.

The names of Dr. J. W. Meredith, of Salem, and Dr. G. A. Miller, of Portland, were presented for membership. The gentlemen named were elected.

By request, Dr. Thompson read the constitution.

Dr. Fiske was introduced to the Society, and in accordance with previous invitation, addressed the Society at some length on "The Effect of Disease in the Body upon the Health and Development of the Teeth."

At the conclusion of Dr. Fiske's essay, Dr. Gray, of Albany, presented a paper on "Dental Therapeutics."

It was resolved that the afternoon session be occupied with the reading of papers on the different theses assigned to the members. The Society then adjourned to 2 P. M.

The Society met at 2 P. M, and was called to order by the President, Dr. Hatch.

Reading of Theses being in order, Dr. Cardwell presented a paper on the "Degeneracy of the Teeth."

Dr. Glenn read an essay on "Dental Irregularities and their Correction," and illustrated his subject with the case of a young lady whose teeth he had corrected.

Dr. Thompson presented a thesis on "Dentistry; its History, Present Status, Claims and Relations."

Dr. Welch offered a paper on the "Cause and Treatment of Loose Teeth".

Discussion of Theses being in order, Dr. Glenn asked for information on the treatment of alveolar abscess. He had applied arsenic occasionally to obtund dentine, and had killed teeth. Abscess followed, the teeth turning dark, and would like to know what method of treatment would be most efficacious in such cases.

Dr. Thompson briefly described a method which he had used in many instances, with success. He first made a thorough opening through the alveolar process, applied remedial agents and continued them until sucessful.

Dr. Miller wanted to know something about bleaching dead teeth.

Dr. Hatch, in answer to the last named gentleman, said that he had used Labarraque's solution and prepared chalk.

In the regular order of discussion, the subject of exposed pulps was presented for debate, and thoroughly discussed by the members present.

Dr. Cardwell had tried the different methods as they came up, and had concluded that no rule could have universal application. Each case must suggest the practice. He had sometimes capped the nerve with a non-conductor, using a covering of os-artificiel, which treatment has frequently succeeded. He thought it best, when possible, to save the nerve. In his practice he had often found mouths which would not tolerate dead teeth, the decay in such instances producing abscess.

Dr. Fiske spoke briefly of the electro-cautery in obtunding sensibility and removing unhealthy growth.

Dr. Carpenter, in behalf of the Medical Department of Willamette University, offered the Society either one or two chairs in that Department.

On motion, the Society adjourned to meet at 10 o'clock, A.M.

SECOND DAY, MORNING SESSION.

The Society met at 10 A. M., the President, Dr. Hatch, in the chair.

The roll was called, when the following members responded: Drs. W. F. Thompson, J. R. Cardwell, J. H. Hatch, J. G Glenn, Jno. Welch, and G. A. Miller, of Portland; L. S. Skiff, J. W. Meredith, and T. Nicklin, of Salem; G. W. Gray, of Albany; and E. W. Biddle of Corvallis.

The minutes of the previous meeting were read and approved.

Dr. Cardwell then proposed Dr. H. Smith for membership. On motion the ballot was had, and he was duly elected.

Drs. Thompson and Gray were appointed a committee to wait on Dr. Smith and notify him of his election.

A communication was read from Dr. S. H. Roberts, of San Francisco, requesting to be made an honorary member. The ballot was had and he was duly elected.

Dr E. R. Fiske was elected an honorary member by acclamation.

Dr. Gray moved to consider the proposition of H. Carpenter, M. D., Dean of the Department of Medicine of Willamette University, to create two chairs in Dentistry in connection with their faculty.

Dr. Hatch presented the following:

*Resolved*, That the Oregon State Dental Society offer their hearty co-operation in the matter of establishing a Dental Department in connection with the Willamette University, and recommend to the consideration of the Trustees of said University, the names of Drs. William F. Thompson and George W. Gray as suitable persons to fill those chairs. Carried.

A communication was received from W. H. Watkinds, inviting the Society to visit the State Prison in a body. On motion accepted.

On motion,

*Resolved*, That the thanks of this Society be tendered to the City Council for use of Council Chamber. Carried.

Adjourned to meet at 2 P.M.

AFTERNOON SESSION.

The Society met at 2 o'clock P. M., Dr. Skiff in the chair.

On Motion, Dr. Miller was elected Secretary *pro tem.*

Theses for next meeting were announced, viz:

Improvements in Operative Dentistry; Dr. W. F. Thompson.

Absorption of Alveolar Processes—Cause and Treatment; Dr. J. R. Cardwell.

Relative Value of the Different Materials Used as a Base for Artificial Teeth; Dr. L. S. Skiff.

Neuralgia—Its Cause and Treatment; Dr. T. L. Nicklin.

Alveolar Abscess, and Treatment; Dr. G. A. Miller.

Dental Pathology and Therapeutics; Dr. Geo W. Gray.

Duties of the operator to his patients and himself; Dr. J. G. Glenn.

Professional Ethics; Dr. G. H. Chance.

Mechanical Dentistry; Dr. H. Smith.

Causes of irregularity of the teeth, and best method of correcting the same; Dr. J. Welch.

Deciduous teeth and their treatment; Dr. E. W. Biddle.

Extracting of teeth and treatment; Dr. J. W. Merideth.

Sensitive dentine and treatment; Dr. J. H. Hatch.

After the subject of sensitive dentine had been appointed to Dr. Hatch, Dr. Glenn desired to hear the views of the different members concerning their treatment of the same.

Dr. Smith recommends carbolic acid.

Dr. Welch recommended sharp instruments and a steady hand.

Dr. Miller reported his treatment with carbolic acid and nitrate of silver, had some failures.

Dr. Skiff recommended dietic treatment, by requesting his patients to refrain from the use of acids a few days previous to the operation.

Dr. Biddle filled with os-artificiel or Hill's stopping until sensitiveness subsided, removed and filled with gold.

On motion, the Society adjourned to visit the State Penitentiary, by invitation previously extended. Adjourned to meet at 8 o'clock P. M.

EVENING SESSION.

Meeting called to order, Dr. Skiff in the chair, when the following names were presented, balloted for, and elected members of the Society, viz: Dr. E. O. Smith, of Albany, and Dr. W. Kohler, of Portland.

On motion, a vote of thanks was tendered E. R. Fiske, M. D. for his excellent paper, and a copy requested for publication in the Dental Journals.

After discussing various topics of interest to the profession, the Society adjourned to meet at Portland on the last Wednesday in March, 1874.

J. R. Cardwell,
*Recording Secretary.*

